# Effects of Telemedicine on Obese Patients With Non-alcoholic Fatty Liver Disease: A Systematic Review and Meta-Analysis

**DOI:** 10.3389/fmed.2021.723790

**Published:** 2021-08-19

**Authors:** Surasak Saokaew, Sukrit Kanchanasurakit, Chayanis Kositamongkol, Kanyanat Chaiyo, Thirada Jirapisut, Narakorn Aomsin, Pit Leewongsakorn, Nathorn Chaiyakunapruk, Pochamana Phisalprapa

**Affiliations:** ^1^Center of Health Outcomes Research and Therapeutic Safety (Cohorts), School of Pharmaceutical Sciences, University of Phayao, Phayao, Thailand; ^2^Unit of Excellence on Clinical Outcomes Research and IntegratioN (UNICORN), School of Pharmaceutical Sciences, University of Phayao, Phayao, Thailand; ^3^Unit of Excellence on Herbal Medicine, School of Pharmaceutical Sciences, University of Phayao, Phayao, Thailand; ^4^Division of Pharmacy Practice, Department of Pharmaceutical Care, School of Pharmaceutical Sciences, University of Phayao, Phayao, Thailand; ^5^Biofunctional Molecule Exploratory Research Group, Biomedicine Research Advancement Centre, School of Pharmacy, Monash University Malaysia, Kuala Lumpur, Malaysia; ^6^Novel Bacteria and Drug Discovery Research Group, Microbiome and Bioresource Research Strength, Jeffrey Cheah School of Medicine and Health Sciences, Monash University Malaysia, Kuala Lumpur, Malaysia; ^7^Division of Ambulatory Medicine, Department of Medicine, Faculty of Medicine Siriraj Hospital, Mahidol University, Bangkok, Thailand; ^8^Department of Pharmacotherapy, College of Pharmacy, University of Utah, Salt Lake City, UT, United States

**Keywords:** non-alcoholic fatty liver disease, obesity, telemedicine, systematic review, meta-analysis

## Abstract

**Background:** Little is known of the efficacy of telemedicine on the clinical outcomes of the high-risk group of non-alcoholic fatty liver disease (NAFLD) patients, such as those with obesity. This study aimed to determine the effects of telemedicine vs. usual care for the management of obese patients with NAFLD.

**Methods:** Literature searches were performed from inception to 1st June 2021 in the following databases: Cochrane CENTRAL, ScienceDirect, PubMed, and Scopus. Prospective trials assessed the effects of telemedicine on obese patients with NAFLD were included. The outcomes of interest were alanine aminotransferase (ALT), aspartate aminotransferase (AST), triglyceride, high-density lipoprotein cholesterol levels, and body mass index, which were reported as weighted mean difference (WMD) with 95% confidence interval (CI).

**Results:** Four studies were examined in the systematic review, one was excluded from the meta-analysis due to an inappropriate group-comparison. In all, 285 obese patients with NAFLD were included in the meta-analysis (70% of those received telemedicine intervention). The mean ages of the patients in the telemedicine and usual-care groups were 51.78 ± 5.91 and 47.30 ± 8.14 years, respectively. Telemedicine significantly decreased ALT levels compared with usual care (WMD = −18.93 U/L [95%CI: −25.97, −11.90]; *I*^2^ = 53.8%), and it significantly decreased AST levels (WMD = −10.24 U/L [95%CI: −13.43, −7.05]; *I*^2^ = 0.0%). However, telemedicine did not show significant benefits for the remaining outcomes.

**Conclusion:** Compared with usual care, telemedicine significantly reduced the AST and ALT levels of obese patients with NAFLD. Further long-term studies with clinical endpoints are needed to determine the best characteristics of telemedicine and to confirm the benefits.

**Systematic Review Registration:** PROSPERO [CRD42020207451].

## Introduction

Non-alcoholic fatty liver disease (NAFLD) is one of the diagnoses that indicate a risk of developing worsened health outcomes. The prevalence of NAFLD ranges from 6 to 33% worldwide (1–3) and its incidence is rising in every region of the world (2). In Europe, NAFLD has been reported to involve about 52 million people annually, with a disease-management cost of around €35 billion per annum (4). Patients with NAFLD are at risk of developing not only chronic liver illnesses, but also other, non-communicable, chronic diseases such as those extra-hepatic manifestations, including cardiovascular diseases and chronic kidney disease, in the future (2, 5). The all-cause mortality rate of NAFLD patients is higher than that for the general population (6, 7). Additionally, there is evidence that NAFLD patients with central obesity have a higher risk of overall and cardiovascular disease-related mortalities than non-obese NAFLD patients (7).

A definite treatment for NAFLD patients has not yet been established, and most current pharmacological treatment trials have had insufficient power to show significant benefits for NAFLD patients (8). Lifestyle modification (diet, exercise, and weight reduction) has been advocated for use in the management of NAFLD patients, and it has been reported to be effective in reducing hepatic steatosis (1). An economic evaluation also demonstrated that weight reduction could not only prevent cirrhosis and hepatocellular carcinoma occurrences, but was also a cost-saving strategy, making it the most appropriate management approach for NAFLD patients (9). However, patient adherence to lifestyle changes is critical because a long time period is required not only to permanently change routine behavior but to achieve the treatment goal. To optimize patient adherence, an intervention such as telemedicine might prove to play a vital role.

Telemedicine is the integrated use of communication technology and health information with the aim of bettering patients' health outcomes by improving the availability of medical information and treatment accessibility. Telemedicine allows healthcare professionals to utilize advanced telecommunication services in order to provide medical services to patients (10, 11). Moreover, it has proven efficacy in improving patients' health outcomes for asthma, chronic obstructive pulmonary disease, heart failure, hypertension, and type 2 diabetes mellitus (12–14).

The COVID-19 pandemic highlighted that the development and implementation of telemedicine is key to maintaining good medical care without requiring patients to unnecessarily visit high infection-risk places—like hospitals. However, the conclusive effects of telemedicine for obese patients with NAFLD are yet to be established. This systematic review and meta-analysis therefore set out to determine the effects of telemedicine for obese patients with NAFLD.

## Methods

This article was performed and reported in accordance with the Preferred Reporting Items for Systematic Reviews and Meta-analyses (PRISMA) statement (15). This study was registered in PROSPERO (registration number: CRD42020207451).

### Search Strategy

The search was performed, without language restriction, from inception to 1st June 2021 using four major electronic databases: Cochrane CENTRAL, ScienceDirect, PubMed, and Scopus. The search terms were “Non-alcoholic fatty liver disease,” “NAFLD,” AND “Telemedicine.” The literature search was limited to research on humans. The search algorithms are provided in Supplementary Table 1.

### Study Selection and Eligibility Criteria

The inclusion criteria for the systematic review were all prospective studies in the 4 databases that had investigated the effects of telemedicine on the surrogate outcomes of obese patients with NAFLD. Obesity was defined as per each study's inclusion criteria, given that the cutoff BMI to indicate obesity varies by ethnicity. Since current practice rarely uses a liver biopsy as a diagnostic method for NAFLD, our inclusion criteria were not limited to those studies with biopsy-proven NAFLD patients. Instead, studies were acceptable if they had enrolled NAFLD patients who had been diagnosed with a reliable method, such as an imaging test. However, the included studies were required to have measured the surrogate outcomes of interest: aspartate aminotransferase (AST), alanine aminotransferase (ALT), body mass index (BMI), triglyceride (TG), and high-density lipoprotein cholesterol (HDL-C) levels.

A study was excluded if it was a review article or meta-analysis, news report, letter, poster presentation, book, or documentation. In addition, papers for which only the abstract was accessible were excluded, as were non-human studies and work that recruited participants under 18 years of age. Duplicated studies were also removed during the review process.

As to the meta-analysis, studies were excluded if they did not perform usual care (as a control group) to compare the pooled effects of telemedicine and usual care. Furthermore, included studies were required to measure the results required for analysis of the different outcomes of the 2 care strategies. On the other hand, our eligibility criteria specified no limitations on the characteristics or durations of the telemedicine interventions.

### Data Extraction

Data from the studies were independently retrieved by five authors (SK, KC, TJ, NA, and PL) using a standard extraction form. The data items were first author; publication year; country of study; study setting; study area (i.e., urban vs. rural); study type; number and characteristics of participants; NAFLD diagnostic method; details of intervention and control; and outcome measurements. The categorization of the telemedicine interventions was based on their proposed activities and their involvements in the case management, consultation, education, monitoring, and reminding processes.

### Quality Assessment

The included studies were independently assessed for their methodological quality by the five aforementioned authors using the Cochrane Risk of Bias tool (version 2.0). The publication bias was not assessed as only a small number of studies were reviewed and pooled.

### Data Analyses

A qualitative synthesis was conducted by summarizing the characteristic and outcomes of included studies. In addition, the attributes and features of telemedicine implemented in each study were extracted, described, and categorized in to 2 main types of communication, i.e., one-way and two-way communications. The results were reported in descriptive text and tables.

A meta-analysis was performed to estimate the effects of telemedicine compared with usual care. Weighted mean difference (WMD) and 95% confidence interval (CI) were used to calculate and compare those effects. The heterogeneity of the studies was assessed using the *I*-squared (*I*^2^) statistical test (16). Subgroup analyses were also performed of the study designs (randomized controlled trial [RCT] and non-RCT) and intervention types (two-way and one-way communication).

## Results

**Figure 1:**
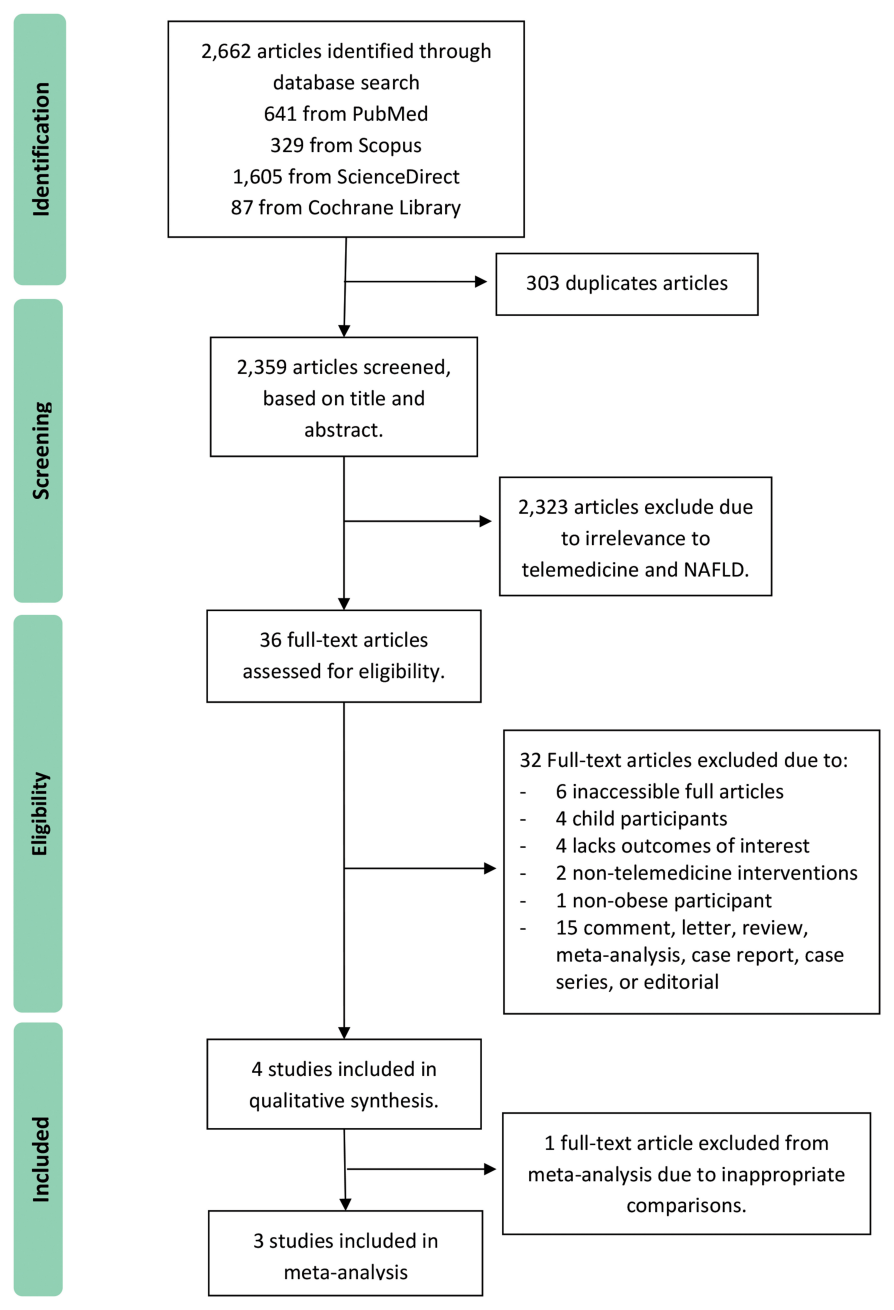
PRISMA flow diagram.

The initial database search yielded 2,662 articles, of which 303 duplicates were removed. The rest were screened via their titles and abstracts, which led to the removal of another 2,323 because of their irrelevance to telemedicine and NAFLD. The remaining 36 articles were fully reviewed for their eligibility. That resulted in 32 being excluded from the systematic review due to the reasons described in Figure 1. In addition, another article was excluded from the meta-analysis due to inappropriate comparisons (17). A total of 4 studies (17–20) were reported in the systematic review results, and 3 studies (18–20) were included in the quantitative synthesis. A Preferred Reporting Items for Systematic Reviews and Meta-Analyses flow diagram is presented at Figure 1.

### Systematic Review

All of the included studies were performed at University hospitals. Three of the four studies were conducted in western countries (17, 19, 20); the fourth was carried out in Western Asia (18). In addition, three (17–19) (75%) were conducted in urban areas, but one (20) (25%) did not identify the study area. Half of the studies were randomized controlled trials (RCTs) (18, 19), while the other half were non-RCTs (17, 20). The characteristic of the included studies is listed in Table 1. A total of 5 patterns of supportive management for NAFLD patients were performed, and these were able to be categorized into two types of communication: one-way and two-way. One-way communication involved text messaging (19), whereas two-way communication comprised telephone (18), on-site education (20), and web-based interventions (17, 20). The number of healthcare professionals involved in the telemedicine initiatives varied among the studies. Physicians were involved in all studies (17–20). Nutritionists/dietitians took part in two of the studies (17, 18). Nurses (20) and psychologists (17) were, each, involved in two separated studies. A variety of medical supportive management approaches were incorporated in the telemedicine activities: self-management support [2 studies (17, 20)]; monitoring patients' health status [4 studies (17–20)]; interactive communication [3 studies (17, 18, 20)]; action plan provision [3 studies (17–19)]; and education support [4 studies (17–20)]. No study utilized a reminding strategy. The duration between the provision of the individual telemedicine services ranged from daily to once a month, while the duration of the studies themselves ranged from 3 to 24 months. The features of the telemedicine initiatives and their supportive management protocols are detailed in Table 2. The results of the surrogate outcomes (such as weight reduction, liver enzyme decrease, and physical activity increase) of the NAFLD patients receiving telemedicine support were better than, or equal to, those of the usual-care or on-site interventions. A study by Fard et al. (18) demonstrated that telephone intervention provided by a nutritionist and a physician significantly decreased ALT and AST levels compared with those of a usual-care group (*P* < 0.001). A study by Axley et al. (19) investigated the effects of text-message management on ALT, AST, TG, HDL-C, and BMI levels. They found that the strategy significantly improved ALT and AST relative to usual care at 6 months (*P* < 0.05), but TG, HDL-C, and BMI were not affected by the intervention. Research by Gomez et al. (20) compared the effects of providing education classes on-site vs. via the Internet, and then compared both approaches with usual care. Their work revealed that both education strategies improved ALT, AST, TG, HDL-C, and BMI levels relative to usual care at 12 months (*P* < 0.01). However, the study by Gomez et al. did not report the differences between the outcomes of the on-site and web-based education approaches. This is because the 2 education groups were combined for analysis purposes, given that there were no significant differences in the values of the biochemical markers of those two groups at baseline and the conclusion of the 1-year study (21). Research by Mazzotti et al. concluded that an interactive, web-based intervention for obese patients with NAFLD was not less effective than a group-based lifestyle modification program in terms of ALT and TG reductions. Nevertheless, the effect of the web-based program on weight reduction was significantly better than that of the group-based program (17).

**Table 1:** Characteristics of the included studies.

**References**	**Country**	**Setting**	**Studied area**	**Study type**	**Sample size** **(intervention vs. control)**	**Patient characteristics**				**Intervention**	**Control**	**Duration (months)**
						**Age** ^*^ **(year)**	**Male (%)**	**NAFLD diagnosis method**	**DM (%)**			
Fard et al. (18)	Iran	Single school of Nursing and Midwifery	Urban	RCT	30 vs. 30	Intervention 40.3 Control 38.3	76.3%	Ultrasound	0%	Telephone	Usual care	3
Axley et al. (19)	USA	Department of Internal Medicine, University of Texas Medical	Urban	RCT	13 vs. 17	Intervention 54 ± 2.7 Control 52 ± 2.3	33%	Ultrasound, elevate liver enzyme	Intervention 38% Control 30%	Text messaging	Usual care	6
Vilar-Gomez et al. (20)	USA	4 Universities	NA	Non-RCT	157 vs. 38	Intervention 53.8 ± 8.4 Control 52.3 ± 9.5	37%	Non-invasive scores	100%	On-site education classes or via web-based	Usual care	12
Mazzotti et al. (17)	Italy	Unit of Metabolic Diseases and Clinical Dietetics, University of Bologna	Urban and rural	Non-RCT	278 vs. 438	Intervention web-treated 46.0 ± 11.5 Intervention group-treated 55.1 ± 12.3	53.5%	Ultrasound	Intervention web-treated 21.6% Intervention group-treated 40.6%	Web-based program	Group-base program	24

*DM, diabetes mellitus; NA, not available; NAFLD, non-alcoholic fatty liver disease; RCT, randomized controlled trial; USA, United States of America*.

* *Values are presented as mean ± standard deviation*.

**Table 2:** Features of telemedicine and the supportive management protocols of the included studies.

**References**	**Type of telemedicine**	**HCP's involvement/ device**	**Medical supportive managements**						**Duration of period between services**
			**Self-management support**	**Monitoring patients' health status**	**Interactive communication (Two-way communication)**	**Action plan provision**	**Education support**	**Reminding strategy**	
Fard et al. (18)	Telephone	Nutritionist, Physician/ telephone	-	Patients were assessed evaluating the adherence to diet and physical activities.	Yes	Yes	Patients were provided dietary and physical activities education.	-	Twice a week during the first month and once a week in the second and the third months
Axley et al. (19)	Text message	Physician/ mobile	-	Patients were also asked about the challenges they face in making healthy diet changes and were provided tips for overcoming those specific challenges (e.g., not enough time, cost of healthy food and lack of knowledge).	-	Yes	Patients received the messages provided education on different domains including nutrition, exercise, and stress management.	-	1 week
Vilar-Gomez et al. (20)	On-site education classes (*n* = 136) or via web-based (*n* = 126)^*^	Physician or nurse practitioner/ web-based	Patients can select between two different educational modes; either via on-site education classes or via web-based	Patients were monitored glycemic and ketosis status through patient reported daily blood glucose and blood beta-hydroxybutyrate over the year.	Yes	-	Patients' dietary modifications included restricting total carbohydrate and fat, adequate intake of minerals, fluids and non-starchy vegetables	-	Daily monitoring for 1 year. On-site education was held weekly for first 12 weeks, bi-weekly for next 12 weeks, and monthly for 6 months. Recorded contents in web-based education could be independently accessed through the application.
Mazzotti et al. (17)	Web-based program	Physicians, dietitians, and psychologist	The individual sessions may be repeated without limitations.	Patients may interact with the clinical center offline, by sending food diaries or asking questions via specific tools.	Yes	Yes	The patients are provided with a series of 25–35 slides per sessions, with texts read by a voiceover and figures to support the text.	-	Sessions could be repeated without limitations. Outcomes were measured every 6 months

*HCP, healthcare professional*.

* *No significant differences were observed in the biochemical markers of the 2 groups of patients for the different modes of education*.

### Meta-Analysis: the Pooled Effects of Telemedicine

Of the 4 studies, one conducted by Mazzotti et al. (17) was excluded from the meta-analysis because it did not compare the effects of telemedicine with usual care (Table 3). Mazzotti el al. compared the outcomes of patients who underwent telemedicine intervention, i.e., web-based program, with those patients participated in group-based program instead of usual care. Their group-based intervention provided more intensive strategy of NAFLD management such as five 120-min weekly sessions that chaired by multidisciplinary team. Psychologist was involved in one of the sessions which was a motivational session to stimulate weight reduction maintenance that usual care in other studies did not mention about. In all, the meta-analysis included 285 patients (18–20), 200 of whom were in the telemedicine group, with the remaining 85 in the control (i.e., usual care) group. The mean ages of the patients ranged from 38 to 54 years; the pooled average ages of the patients in the intervention and control groups were 51.78 ± 5.91 and 47.30 ± 8.14 years, respectively. The pooled baseline BMIs of the intervention and control groups were 37.78 ± 4.62 and 34.07 ± 5.09 kg/m^2^, respectively.

**Table 3:** Summary of study outcomes.

**References**	**Interventions**	**Sample size (*N*)**	**Outcomes** ^*^ **(baseline vs. final visit)**				
			**ALT (U/L)**	**AST (U/L)**	**TG (mg/dL)**	**HDL-C (mg/dL)**	**BMI (kg/m** ^**2**^ **)**
Fard et al. (18)	Usual care + telephone	30	80.30 ± 38.59 vs. 36.60 ± 19.27	46.83 ± 17.64 vs. 28.80 ± 9.85	NA	NA	28.8 ± 4.32 vs. 26.36 ± 3.95
	Usual care	30	63.52 ± 23.67 vs. 65.03 ± 28.68	38.52 ± 12.90 vs. 39.79 ± 15.33	NA	NA	28.5 ± 4.28 vs. 27.92 ± 4.19
Axley et al. (19)	Usual care +text messages	13	44 ± 6 vs. 32 ± 3	46 ± 6 vs. 37 ± 4	184 ± 34 vs.167 ± 35	40 ± 4 vs. 38 ± 4	39 ± 2 vs. 38 ± 3
	Usual care	17	57 ± 12 vs. 51 ± 11	47 ± 7 vs. 47 ± 8	194 ± 37 vs. 153 ± 25	40 ± 3 vs. 40 ± 3	36 ± 2 vs. 37 ± 2
Vilar-Gomez et al. (20)	Usual care + interventions	157	39.2 ± 25.4 vs. 25.8 ± 15.4	28.5 ± 17.6 vs. 20.7 ± 8.5	196.3 ± 120.2vs. 152.8 ± 130.6	43.2 ± 13.8 vs. 49.9 ± 16.5	39.4 ± 7.2 vs. 34.3 ± 6.5
	Usual care	38	40.7 ± 23.4 vs. 38.6 ± 24.9	32.7 ± 26.1 vs. 30.7 ± 20.5	197.4 ± 100.5vs. 214.2 ± 149.8	41 ± 11.3 vs. 37.2 ± 11.4	37.6 ± 6.9 vs. 38.2 ± 8.2

*ALT, alanine aminotransferase; AST, aspartate aminotransferase; BMI, body mass index; HDL-C, high-density lipoprotein cholesterol; NA, not available; TG, triglyceride*.

* *Values are presented as mean ± standard deviation*.

### Primary Outcomes

**Figure 2:**
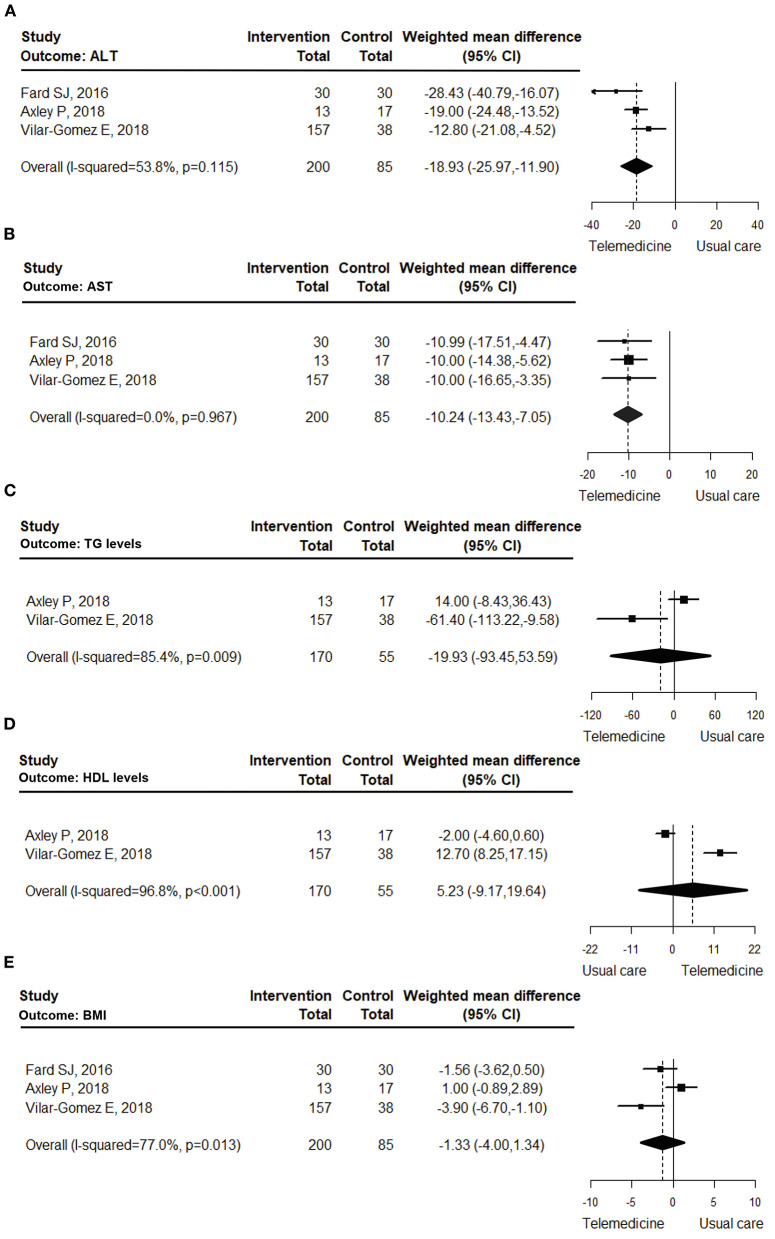
Forest plot of the effects of telemedicine compared with usual care on the ALT **(A)**, AST **(B)**, triglyceride **(C)**, HDL-C **(D)**, and BMI **(E)** levels of obese patients with NAFLD.

**ALT:** The meta-analysis pooled the outcomes of 3 studies (18–20) that investigated the effects of telemedicine on the ALT levels of obese patients with NAFLD. The results indicated that the telemedicine interventions contributed to significantly decreased ALT levels in obese patients with NAFLD relative to those receiving usual care (WMD = −18.93 U/L [95%CI: −25.97, −11.90]; *I*^2^= 53.8%; Figure 2A). In the subgroup analyses, both the two-way and one-way communication interventions significantly reduced the ALT levels (two-way communication (18, 20): WMD = −19.91 U/L [95%CI: −35.17, −4.66]; *I*^2^= 76.4%; and one-way communication (19): WMD = −19.00 U/L [95%CI: −24.48, −13.52]). The intervention-favored results were apparent in the RCT studies (18, 19) (WMD = −22.02 U/L [95%CI: −30.64, −13.40]; *I*^2^ = 46.5%) and the non-RCT study (20) (WMD = −12.80 U/L [95%CI: −21.08, −4.52]). The subgroup analysis results are presented in Supplementary Table 2.

### Secondary Outcomes

**AST:** The meta-analysis investigated 3 studies (18–20) to determine the effects of telemedicine on AST levels. The results revealed that the telemedicine interventions significantly decreased the AST levels of the obese patients with NAFLD compared with those undergoing usual care (WMD = −10.24 U/L [95%CI: −13.43, −7.05]; *I*^2^= 0.0%; Figure 2B). In the subgroup analysis, both the two-way and one way-communication interventions resulted in significant reductions in AST levels (two-way communication (18, 20): WMD = −10.50 U/L [95%CI: −15.16, −5.85]; *I*^2^= 0.0%; and one-way communication (19): WMD = −10.00 U/L [95%CI: −14.38, −5.62]). As to the RCT studies (18, 19), telemedicine showed WMDs between the groups of −10.31 U/L ([95%CI: −13.94, −6.67]; *I*^2^= 0.0%), while the non-RCT study (20) showed a WMD of −10.00 U/L [95%CI: −16.65, −3.35]. The subgroup analysis results are detailed in Supplementary Table 2.

**TG:** The 2 studies (19, 20) that investigated the effects of telemedicine on TG levels were included in the meta-analysis. Their pooled results revealed that telemedicine did not significantly decrease the TG levels of the obese patients with NAFLD (WMD = −19.93 mg/dL [95%CI: −93.45, 53.59]; *I*^2^= 85.4%; Figure 2C).

**HDL-C:** The 2 studies (19, 20) that examined the effects of telemedicine on HDL-C levels were analyzed. Their pooled results demonstrated that telemedicine did not significantly increase the HDL-C levels of the obese patients with NAFLD (WMD = 5.23 mg/dL [95%CI: −9.17, 19.64]; *I*^2^= 96.8%; Figure 2D).

**BMI:** Three studies (18–20) exploring the effects of telemedicine on BMI were reviewed and pooled. The pooled results showed that telemedicine did not significantly lessen the BMI of the obese patients with NAFLD (WMD = −1.33 kg/m^2^ [95%CI: −4.00, 1.34]; *I*^2^= 77.0%; Figure 2E). Moreover, the subgroup analysis results indicated that telemedicine provided a significantly decreased BMI only for the two-way communication intervention (18, 20) (WMD = −2.53 kg/m^2^ [95%CI: −4.79, −0.27]; *I*^2^= 42.6%) and the non-RCT study (20) (WMD = −3.90 kg/m^2^ [95%CI: −6.70, −1.10]). The subgroup analysis results are in Supplementary Table 2.

### Quality Assessment

All 3 studies in the meta-analysis (18–20) had a negligible risk of bias in missing outcome data and reporting bias, but a substantial risk of performance bias blinding. Of the two non-RCTs in systematic review (17, 20), one (17) that was excluded from the meta-analysis had risks of concealment, blinding, and assessment. The risk-of-bias assessment are shown in Supplementary Figure 1.

## Discussion

Despite a few studies having been conducted to evaluate the use of telemedicine for NAFLD management, the conclusive effects of telemedicine are still to be clearly established. This is the first systematic review and meta-analysis to investigate the effects of telemedicine on the ALT, AST, TG, HDL-C, and BMI levels of obese patients with NAFLD. The addition of telemedicine was found to decrease ALT and AST levels better than that achieved via usual care alone. This evidence may guide the discovery or development of strategies that could work in conjunction with telemedicine to improve the management of NAFLD in the future. Regarding the effects of telemedicine on the management of other diseases, a systematic review and meta-analysis (22) investigated its impact on weight reduction and clinical-outcome improvement in obese patients. That work revealed that using a telephone produced a significant difference in the body weight loss achieved by members of the intervention and control groups. However, our meta-analysis showed that telemedicine did not significantly lessen the BMI of the obese patients with NAFLD.

The effects of telemedicine might depend on the characteristics and duration of the intervention. We attempted to elucidate the impact of those factors via the subgroup analyses, given that all 4 studies were conducted with various intervention durations and differing time points for outcome assessments. Unfortunately, as the number of relevant studies was too small, the only subgroup analyses that could be carried out related to evaluation of the treatment effects on ALT, AST, and BMI levels in patient subgroups defined by the study design and intervention type. The results of the subgroup analyses did not alter the results of the main analysis. Nevertheless, the results of the subgroup analyses should be interpreted with caution as they included two RCTs which examined different communication types. One critical issue is that NAFLD is a slow-progressing disease. Hence, in order to observe the final endpoints (e.g., cirrhosis, cancer, and non-liver-related outcomes), future studies should consider using a long follow-up period.

Although the reported average age of the patients in each included study was more than 18 years, the provided detail of those studies was not sufficient for us to determine if any non-adult patient was included in the cohort. This rigid inclusion of the current work is mentioned because we believe that the care of the adults and non-adults is distinctively different. Adolescents with NAFLD may need some help from their caregivers to maintain their proper diet and to manage their routine exercise, whereas adults are usually capable of self-care. Including adolescents in our review may create clinical heterogeneity from the disparity of patient groups. Furthermore, the effects of telemedicine for adolescent patients might be altered due to the additional support they receive from their caregivers, which is considered a non-telemedicine care strategy. Another point that we need to mention is that the study by Gomez et al. (20) consisted of both on-site and web-based education interventions. However, the multiple comparisons conducted by those researchers confirmed that the use of the on-site and web-based strategies resulted in no differences in their corresponding baseline and outcome biochemical markers.

The main limitations of this study are its small number of included studies and patients. These factors reduce the generalizability of the findings and make it difficult to recommend the use of tele-interventions in clinical practice. Also, none of those patients was biopsy-proven NAFLD. Nevertheless, they were diagnosed by standard imaging technique (i.e., ultrasound) and non-invasive prediction score (i.e., NAFLD liver fat score) which was proven to have a sensitivity of 86% and a specificity of 71% (23, 24). All patients with any secondary cause of hepatitis and those with significant alcohol intake were excluded. In addition, we were not able to perform the subgroup analyses of the intervention and follow-up durations because of the restricted number of included studies. This is unfortunate as the variations in the lengths of the interventions and follow-up periods of the included studies was suspected to be associated with the efficacy of telemedicine that was represented through the surrogate outcomes. Healthcare professionals who are considering the implementation of telemedicine interventions should evaluate whether their settings are comparable with those in the 4 studies. If they do decide to introduce telemedicine in their practice, they will need to determine their own follow-up period, given the absence of any current evidence for an appropriate intervention length.

In light of the COVID-19 pandemic, telemedicine is taking on an increasingly important role. It changes the traditional patterns of patient care by upgrading patient access to healthcare services, improving health outcomes, and reducing healthcare costs (25, 26). Telemedicine helps to provide patients with clinical education and supportive medical management. However, patients' inability to acquire, accept, or use the related technology could limit the use of telemedicine in practice, especially in developing countries. Although the effects of telemedicine interventions were evaluated by the present study, the challenge is to conduct research that determines the best characteristics of telemedicine for specific patient groups and analyzes the cost-effectiveness of various telemedicine strategies. Moreover, the ethical, legal, economic, and sociocultural aspects need to be considered before developing as well as implementing any strategy. Our findings provide healthcare professionals with current evidence of telemedicine's effects for NAFLD management. These findings can be used to guide the development of suitable telemedicine models for the care of obese patients with NAFLD.

## Conclusion

In conclusions, this systematic review and meta-analysis gathered all relevant evidence and quantified pooled estimates to evaluate the effects of telemedicine on adult obese patients with NAFLD. The use of telemedicine significantly reduce AST and ALT levels in obese patients with NAFLD. Further long-term studies with clinical endpoints are needed to determine the best characteristics of telemedicine and to confirm the clinical benefits.

## Data Availability Statement

The original contributions presented in the study are included in the article/Supplementary Material, further inquiries can be directed to the corresponding author.

## Ethics Statement

The systematic review or meta-analysis is exempt from ethics approval because it is collecting and synthesizing data from previous studies. In addition, patient data is anonymized and data are available in the public domain so that ethical permission is not needed. The authors followed applicable EQUATOR Network (https://www.equator-network.org) guidelines during the conduct of research project.

## Author Contributions

SS, SK, CK, KC, TJ, NA, PL, NC, and PP: study concept and design. SS, SK, CK, KC, TJ, NA, and PL: acquisition of data. SS, SK, CK, KC, TJ, NA, PL, and NC: statistical analysis. SS, SK, CK, KC, TJ, NA, PL, and PP: analysis and interpretation of data and drafting of the manuscript. SS, SK, CK, NC, and PP: critical revision of the manuscript. All authors contributed to the article and approved the submitted version.

## Conflict of Interest

The authors declare that the research was conducted in the absence of any commercial or financial relationships that could be construed as a potential conflict of interest.

## Publisher's Note

All claims expressed in this article are solely those of the authors and do not necessarily represent those of their affiliated organizations, or those of the publisher, the editors and the reviewers. Any product that may be evaluated in this article, or claim that may be made by its manufacturer, is not guaranteed or endorsed by the publisher.
