# Supplementary material for: Effects of Telemedicine on Obese Patients With Non-alcoholic Fatty Liver Disease: A Systematic Review and Meta-Analysis
Source: Front Med (Lausanne). 2021 Aug 19;8:723790. doi: 10.3389/fmed.2021.723790 (PMC8417362; doi:10.3389/fmed.2021.723790)
Supplement: Supplementary file 1 [file Data_Sheet_1.pdf]

FRONTIERS IN MEDICINE

SUPPLEMENTARY DATA

Effects of telemedicine on obese patients with non-alcoholic fatty liver disease: a systematic review and meta-analysis

Surasak Saokaew[1–6], Sukrit Kanchanasurakit[1,4], Chayanis Kositamongkol[7], Kanyanat Chaiyo[4], Thirada Jirapisut[4], Narakorn Aomsin[4], Pit Leewongsakorn[4], Nathorn Chaiyakunapruk[8], Pochamana Phisalprapa[7*]

[1]Center of Health Outcomes Research and Therapeutic Safety (Cohorts), School of Pharmaceutical Sciences, University of Phayao, Phayao, Thailand

[2]Unit of Excellence on Clinical Outcomes Research and IntegratioN (UNICORN), School of Pharmaceutical Sciences, University of Phayao, Phayao, Thailand

[3]Unit of Excellence on Herbal Medicine, School of Pharmaceutical Sciences, University of Phayao, Phayao, Thailand

[4]Division of Pharmacy Practice, Department of Pharmaceutical Care, School of Pharmaceutical Sciences, University of Phayao, Phayao, Thailand

[5]Biofunctional Molecule Exploratory Research Group, Biomedicine Research Advancement Centre, School of Pharmacy, Monash University Malaysia, Bandar Sunway, Selangor Darul Ehsan, Malaysia

[6]Novel Bacteria and Drug Discovery Research Group, Microbiome and Bioresource Research Strength, Jeffrey Cheah School of Medicine and Health Sciences, Monash University Malaysia, Bandar Sunway, Selangor Darul Ehsan, Malaysia

[7]Division of Ambulatory Medicine, Department of Medicine, Faculty of Medicine Siriraj Hospital, Mahidol University, Bangkok, Thailand

[8]Department of Pharmacotherapy, College of Pharmacy, University of Utah, Salt Lake City, Utah, USA

*** Correspondence:**

Pochamana Phisalprapa, M.D., Ph.D.
Division of Ambulatory Medicine, Department of Medicine,
Faculty of Medicine Siriraj Hospital, Mahidol University, Bangkok, Thailand, 10700
Tel: +66 2 419 7190; Fax: +66 2 419 7190
E-mail address: coco_a105@hotmail.com

TABLE LEGENDS

Table S1 Search algorithms
Table S2 Subgroup analyses

FIGURE LEGENDS

Figure S1 Risk of bias

Table S1 Search algorithms

Database	No	Step search algorithm	Items found
Pubmed	#1	Search communications media	298927
	#2	Search telecommunications	88431
	#3	Search telemedicine	28525
	#4	Search telenursing	328
	#5	Search phone	27062
	#6	Search digital	121795
	#7	Search web	102680
	#8	Search mobile	96534
	#9	Search application	719575
	#10	Search non-alcoholic fatty liver disease	14046
	#11	Search ((((((((communications media) OR telecommunications) OR telemedicine) OR telenursing) OR phone) OR digital) OR web) OR mobile) OR application	1299753
	#12	Search (non-alcoholic fatty liver disease) AND (((((((((communications media) OR telecommunications) OR telemedicine) OR telenursing) OR phone) OR digital) OR web) OR mobile) OR application)	**641**
Scopus	#1	Search communications media	6662
	#2	Search telecommunications	496688
	#3	Search telemedicine	33391
	#4	Search telenursing	413
	#5	Search phone	110034
	#6	Search digital	1013250
	#7	Search web	486321
	#8	Search mobile	659779
	#9	Search application	5620186
	#10	Search non-alcoholic fatty liver disease	11977
	#11	Search ((((((((communications media) OR telecommunications) OR telemedicine) OR telenursing) OR phone) OR digital) OR web) OR mobile) OR application	7496202
	#12	Search (non-alcoholic fatty liver disease) AND (((((((((communications media) OR telecommunications) OR telemedicine) OR telenursing) OR phone) OR digital) OR web) OR mobile) OR application)	**329**
Cochrane	#1	Search communications media	34
	#2	Search telecommunications	423
	#3	Search telemedicine	2527
	#4	Search telenursing	55
	#5	Search phone	7214
	#6	Search digital	10571
	#7	Search web	6705
	#8	Search mobile	17413
	#9	Search application	44958
	#10	Search non-alcoholic fatty liver disease	988
	#11	Search ((((((((communications media) OR telecommunications) OR telemedicine) OR telenursing) OR phone) OR digital) OR web) OR mobile) OR application	81559
	#12	Search (non-alcoholic fatty liver disease) AND (((((((((communications media) OR telecommunications) OR	**87**

Database	No	Step search algorithm	Items found
		telemedicine) OR telenursing) OR phone) OR digital) OR web) OR mobile) OR application)	
Science direct	#1	Search communications media	1597
	#2	Search telecommunications	91522
	#3	Search telemedicine	12673
	#4	Search telenursing	122
	#5	Search phone	346853
	#6	Search digital	1042030
	#7	Search web	1140794
	#8	Search mobile	733817
	#9	Search application	5540829
	#10	Search non-alcoholic fatty liver disease	10988
	#11	Search ((((((((communications media) OR telecommunications) OR telemedicine) OR telenursing) OR phone) OR digital) OR web) OR mobile) OR application	6949220
	#12	Search (non-alcoholic fatty liver disease) AND (((((((((communications media) OR telecommunications) OR telemedicine) OR telenursing) OR phone) OR digital) OR web) OR mobile) OR application)	**1605**

Table S2 Subgroup analyses

Characteristics	ALT (U/L)			AST (U/L)			Triglyceride levels (mg/dL)			HDL-C levels (mg/dL)			BMI (kg/m^2)		
	WMD (95% CI)	Heterogeneity		WMD (95% CI)	Heterogeneity		WMD (95% CI)	Heterogeneity		WMD (95% CI)	Heterogeneity		WMD (95% CI)	Heterogeneity	
		I^2 value (%)	*p-value*		I^2 value (%)	*p-value*		I^2 value (%)	*p-value*		I^2 value (%)	*p-value*		I^2 value (%)	*p-value*
**Study design**															
RCT	-22.02 (-30.64, -13.40)	46.5	0.172	-10.31 (-13.94, -6.67)	0.0	0.805	14.00 (-8.43, 36.43)	NA	NA	-2.00 (-4.60, 0.60)	NA	NA	0.25 (-2.75, 2.26)	69.0	0.073
Non-RCT	-12.80 (-21.08, -4.52)	NA	NA	-10.00 (-16.65, -3.35)	NA	NA	-61.40 (-113.22, -9.58)	NA	NA	12.70 (8.25, 17.15)	NA	NA	-3.90 (-6.70, -1.10)	NA	NA
**Communication**															
One-way	-19.00 (-24.48, -13.52)	NA	NA	-10.00 (-14.38, -5.62)	NA	NA	14.00 (-8.43, 36.43)	NA	NA	-2.00 (-4.60, 0.60)	NA	NA	1.00 (-0.89, 2.89)	NA	NA
Two-way	-19.91 (-35.17, -4.66)	76.4	0.039	-10.50 (-15.16, -5.85)	0.0	0.835	-61.40 (-113.22, -9.58)	NA	NA	12.70 (8.25, 17.15)	NA	NA	-2.53 (-4.79, -0.27)	42.6	0.187

ALT, alanine aminotransferase; AST, aspartate aminotransferase; BMI, body mass index; CI, confidence interval; HDL-C, high-density lipoprotein cholesterol; NA, not available; WMD, weighted mean difference

(A)

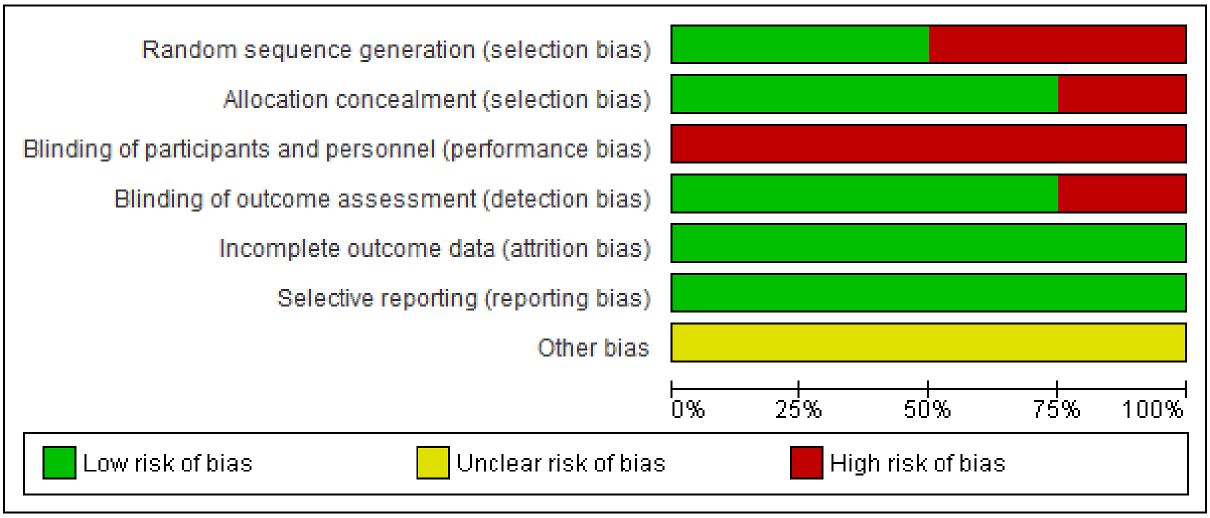

(B)

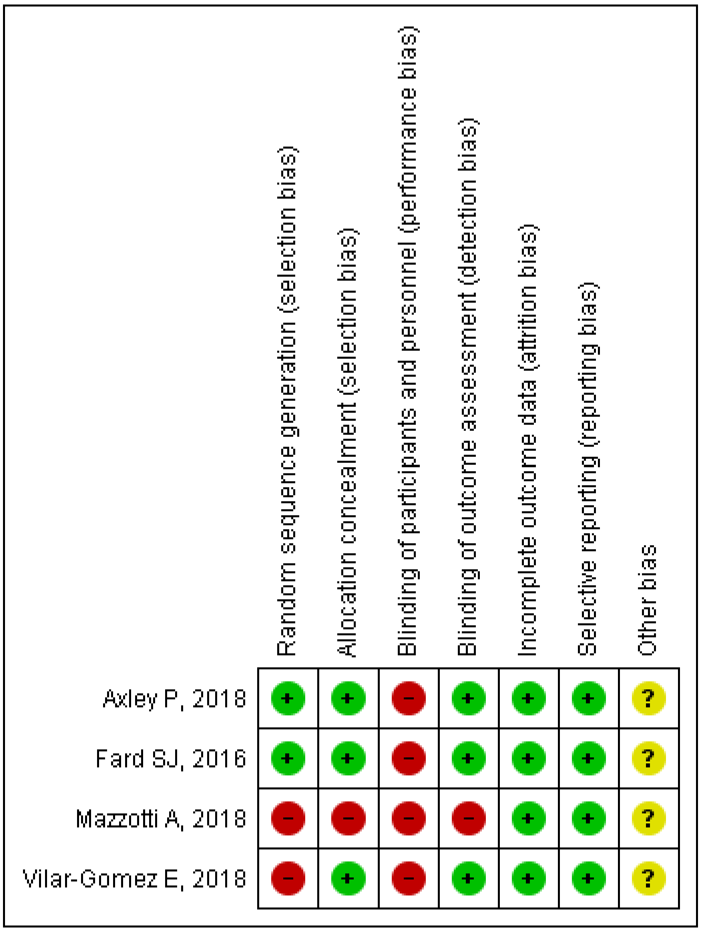

Figure S1 Risk of bias. (A) Risk of bias graph: review authors' judgements on each risk of bias item, presented as percentages across all included studies. (B) Risk of bias summary: review authors' judgements on each risk of bias item for each included study
